# Male partner involvement in HIV testing and counseling among partners of pregnant women in the Delanta District, Ethiopia

**DOI:** 10.1371/journal.pone.0248436

**Published:** 2021-03-17

**Authors:** Haile Chanyalew, Eshetu Girma, Tesfaye Birhane, Muluken Genetu Chanie

**Affiliations:** 1 Delanta District Health Office, Delanta, South Wollo, Ethiopia; 2 School of Public Health, Addis Ababa University, Addis Ababa, Ethiopia; 3 School of Public Health, College of Medicine and Health Sciences, Wollo University, Dessie, Ethiopia; Emory University, UNITED STATES

## Abstract

**Background:**

Only screening a pregnant mother is not satisfactory to prevent mother-to-child transmission of HIV (PMTCT). A male partner’s involvement in HIV testing and counseling is also critical for PMTCT, however, it is one of the biggest challenges in Ethiopia. This study aimed to assess a male partner’s involvement in HIV testing and counseling and associated factors among partners of pregnant women in the Delanta District, Northern Ethiopia.

**Methods:**

A community-based cross-sectional study design was conducted in the Delanta District from March 15 to May 10, 2018. During the study period, 609 male partners were involved. A binary and multiple logistic regression model was used to examine the association between variables.

**Results:**

Out of all, 325 (53.7% at 95% CI: 49.6 to 57.5) of male partners were involved in HIV testing and counseling in the District. Male partners who were living together, ever heard about HIV from health professionals, pregnant women’s antenatal care (ANC) visit, partner visited the PMTCT clinic with wife, and partner and wife discussion before HIV testing and counseling were factors associated with male partner involvement.

**Conclusion:**

The proportion of male partner involvement was found to be low as compared to the national standards. Local health authorities and health care workers need to develop and conduct interventions that help partners with their wife to live together, improve their awareness about HIV and testing, ANC visit by pregnant women, and encourage having home discussion before HIV testing through counseling, by so doing finally raise the level of male partner involvement in HIV testing and counseling.

## Background

Human immunodeficiency virus (HIV) is a major global health challenge affecting approximately 36.7 million people and 1.8 million children [1]. The majority of people living with HIV are located in low- and middle-income countries, with an expected 25.5 million living in sub-Saharan Africa [2]. Ethiopia is one of the top 20 countries affected by HIV in the world. The national adult HIV prevalence was 1.5% (1.9% in women and 1.0% in men). Furthermore, 13,008 children were infected with HIV annually through mother-to-child transmission of HIV (MTCT) [3,4].

Seventy percent of the people globally and 55% of people in Africa knew their HIV status [1]. In Ethiopia, HIV testing and counseling (HTC) uptake by adults was 43% female and only 23% among males in the Amhara region, Ethiopia [5].

Without intervention, the risk of HIV infection from an infected pregnant mother to their unborn child ranges from 25%-35% in the developing countries [6]. The rate of transmission was estimated to be 5%-10% during pregnancy, 15% during delivery, and 5–25% through breastfeeding. However, timely interventions can reduce to 2–5% with the implementation of core Prevention of Mother to Child Transmission of HIV (PMTCT) interventions [7].

Only screening pregnant mothers are not satisfactory for PMTCT. Thus, male partners’ involvement in HTC is critical for MTCT. The magnitude of involvement of male partners attending at least one antenatal care has ranged from 32% to 64.5% in sub-Saharan Africa [8,9], 6% to 58.3% in Ethiopia [10,11]. A health facility-based cross-sectional study conducted in Gondar town revealed that 40.1% of male partners were involved with their pregnant wife for PMTCT [12].

Socio-demographic characteristics, such as age, level of education, relationship status, and employment status were found to be significantly associated with male partner involvement (MPI) in HTC [13,14]. Some of the community-based barriers were socio-cultural in nature and include women’s domain and poor communication between partners that disapprove of male partners engaging in antenatal care activities. This deep-seated perception that antenatal activities are a woman’s responsibility has limited the role of male partners to only providing financial support during pregnancy [8,15–17].

Ethiopia has a national consensus that strives to reduce HIV infection by 90%, identify and put HIV-positive pregnant women on antiretroviral therapy (ART) to 95%. About 90% of people living with HIV know their HIV status through offered targeted HTC and right-based testing by 2020 [18]. Additional targets are zero HIV infection by 2030 and eliminating new HIV infections among infants born from HIV-positive mothers [19].

Male partner involvement in HTC during ANC visit is still a major challenge in the low-and middle-income countries including Ethiopia. Additionally, there is limited data on male involvement in Ethiopia, specifically in Amhara national regional state as well as in the study area [20,21]. Mainly the control of this confronting problem rolled on the hand of the male partner in early screening, diagnosis, and initiation of antiretroviral diseases (ARVs) treatment, and the involvement of their wives. Still detectable numbers of mothers deliver in their homes in Ethiopia. Furthermore, information obtained from the study could serve as baseline data for further research in this study area. Therefore, this study was aimed to assess male partner involvement in HTC and associated factors among partners of pregnant women in the Delanta District.

## Methods and materials

### Study design and setting

A community-based cross-sectional study design was conducted in Delanta District, Northern Ethiopia, from March 15 to May 10, 2018. The estimated size of population of this district was 139,618 with 4705 pregnant women (Delanta District health office report, 2017). The District has 31 rural and 2 urban Kebeles having an area of 126,879 square kilometers. There are 218 health professionals, 55 rural and 1 urban health extension workers, and 6 health centers, 30 health posts, and one primary hospital. All those health centers give HTC and PMTCT services. In the Delanta District, 527 people were living with HIV (PLWHIV) who are on ART [15]. Partners of pregnant women who were living together in the selected Kebeles of the district were interviewed for data collection. But seriously sick respondents were excluded.

### Sample size determination and sampling procedure

The sample size was determined by using a single population proportion formula with the assumptions of 95% level of confidence, 40.1% proportion of male partner’s involvement [12], a margin of error (5%), and design effect = 1.5, possible nonresponse rate = 10% which become 609 sample sizes.

A stratified sampling technique was used to select study participants. Ten out of 31 rural Kebles and one out of two urban Kebeles were selected by using the lottery method. From those selected 11 Kebeles in the District, 1008 male partners of pregnant mothers were obtained. The sampling frame was taken from each health post. Proportional allocation of samples was made to obtain the required sample sizes (n) in each Kebele. Eighty-eight and 521 male partners from urban and rural health posts were selected using the computerized lottery method, respectively. Then male partners were traced to their place of residence using the information obtained from the health post register, and recruit for data collection after obtaining informed consent.

### Data collection measurement

A structured, pretested, and an interviewer-administered questionnaire was used for data collection. It was prepared in English and translated into the local language-Amharic by language experts to check its consistency. Six interviewers (qualified with BSc Midwifery) and three supervisors (qualified with BSc Nurses) competent with local language were recruited and received two days of training on study procedures. The questionnaire was pretested at 5% of the sample in a neighboring district to ensure the validity of the tools.

### Operational definition

**Male involvement on HTC**: this outcome variable was measured by five items adapted from previous studies [8,20]. These are male partners who know ANC appointment, tested for HIV with their wives, discuss antenatal care for HIV tests with wife, provide financial support, and attend ANC with their wives. The involvement score for each respondent ranges from zero (no involvement) to five (involved in all five activities). A total score of 4–5 was considered as a ’high’ male involvement while 0–3 as ’low’ male involvement.

**Knowledge about the route of MTCT**:—when respondents know at least one route of transmission of MTCT of HIV from three questions provided to them to measure their knowledge on the route of MTCT [12].

**Knowledge about PMTCT**:—was measured when respondents know at least one PMTCT related question from the three knowledge measuring items [12].

#### Data processing and analysis

The data were cleaned, coded, and entered into Epi Data software version 3.1. Data were exported and analyzed by using the SPSS version 20 statistical package. Proportion and frequencies were used to describe the study population. The results of the outcome variable measurement were dichotomized into male partner involvement in HTC (yes) and no male partner involvement in HTC (NO). Male partners who responded to four and above male involvement questions were categorized as “yes = 1”, and those who responded to less than or equal to three items were categorized as “no = 0”. On the other hand, by adding questions on the three possible periods of MTCT of HIV, and a male partner reported that at least one question from the three possible periods of MTCT of HIV was categorized as knowledgeable about MTCT. Regarding the wealth indexes of male partners: if they reported their income level is between 0–33 percent classified as poor, 34%-66.67% classified as medium, and more than 67% were classified as high-income levels (wealth index of household, WHO 2015). Binary logistic regression was used to examine the association between the dependent and each independent variable. All variables with *p* < 0.2 on bivariable logistic regression were entered into a multivariable logistic regression model to identify factors independently associated with male involvement in HTC during pregnancy. Enter model was used to select the final independent predictors. The strength of association between the independent and the outcome variables was measured by using odds ratios and 95% confidence interval and p values below 0.05 were declared statistically significant. Multi-collinearity among independently associated variables was checked by multicollinearity diagnostic test variance inflation factor (VIF) in linear regression and none was collinear.

### Ethical consideration

Ethical clearance was obtained from the ethical review committee of Wollo University, College of Medicine, and the health sciences department of public health, and a permission letter was secured from the Delanta district health office. Verbal informed consent from each study participant was obtained. The participants were told about their rights to withdraw from the study at any time, their rights to refuse to give information, and to ask for clarification about the purpose of the study. Confidentiality of the information was assured by omitting names and using unique code identifiers from the questionnaire for privacy participants were interviewed individually in separate places. The data was not accessed by a third person except the principal investigator and was kept confidential and anonymous.

## Results

### Socio-demographic characteristics

Six hundred and five male partners had participated in the study with a response rate of 99.34%. The mean age of male partners of pregnant mothers was 29.88 years. Regarding educational status, 244 (40.3%) were unable to read and write. Five hundred and forty-five (90%) of the participants were living together with their pregnant wife (Table 1).

**Table 1:** Socio-demographic characteristics of partners of pregnant women in Delanta District in Northern Ethiopia, in 2018 (n = 605).

Variables	category	Frequency	percent
Age	20–29 years	314	51.9
	30–39 years	198	32.7
	>40+ year	93	15.4
Residence	Rural	521	86%
	Urban	84	14%
Educational status	Unable to read and write	244	40.3
	Able to read and write	220	36.4
	Primary school	47	7.8
	Secondary	45	7.4
	College and above	49	8.1
Occupation status	Farmer	412	68.1
	Student	33	5.5
	Merchant	75	12.4
	Government Employees	60	9.9
	Daily labor	25	4.1
Living together	Yes	545	90
	No	60	10
Duration of living together (n = 545)	1–5 years	195	36
	6–10 years	166	30.5
	11–15 years	116	21
	16+years	68	12.5
Wealth index	Highest	71	11.7
	Middle	261	43.1
	Lowest	273	45.3

### Comprehensive knowledge on HIV/AIDS, HTC, PMTCT, and perceived risk to acquire the virus

Among the total respondents, 592 (97.9%) ever heard of HIV. The major source of information was from the health care providers (510 (86.1%)), and the least common source was from relatives (157 (26.5%)). About 263 (45.5%) of respondents knew at least two routes of MTCT of HIV and 476 (78.7%) knew the presence of HIV testing and counseling (Table 2).

**Table 2:** Knowledge of partners of pregnant women on HIV/AIDS, HTC, PMTCT in Delanta District, in Northern Ethiopia, in 2018 (n = 605).

Variables	Frequency	percent
Comprehensive knowledge? (n = 605)		
Yes	478	79
No	127	21
**Ever heard of HIV/AIDS (n = 592)**		
Yes	592	100
**Source of information (n = 592)**		
Health professional	510	86.1
Health development army	277	46.8
Mass media	167	28.2
Religious leaders	163	27.5
My wives	162	27.4
Relatives/friends	157	26.5
**The male partner who answered the route of MTCT (n = 549)**		
During labor and delivery	505	83.5
During pregnancy	529	91.7
Breastfeeding	515	89.4
**How many exact timing of MTCT was answered by male partners (n = 577)**		
None	28	4.9
One	151	26.2
Two	263	45.5
Three	135	23.4
Male partners who know the presence of HTC	476	78.7
**A Male partner who knows the method of PMTCT (n = 569)**		
ART	507	89.1
Delivery by Cesarean section	407	71.5
Avoiding breastfeeding	426	74.9
**Do you think you can get the virus? (n = 605)**		
Yes	180	29.8
No	282	46.6
I don’t know	143	23.6
**Reasons for risk (n = 180)**		
Had multiple sexual partners	81	45
Had sexual contact without a condom	57	31.7
Use an unsterile sharp object	42	23.3

### Wives obstetric characteristics and information received from a health care provider

The Majority of the respondents 530 (87.6%) reported that their wives had ANC visits. Only 151 (25%) pregnant mothers had four and above antenatal care visit (Table 3).

**Table 3:** Wives obstetric characteristics and information received from health care provider at Delanta District Northern Ethiopia, 2018 (n = 605).

Variables	Frequency	Percent
**Wives’ number of pregnancies** (n = 605)		
One	70	11.6
Two	113	18.7
Three	243	40.1
Four and above	179	29.6
**Wives Antenatal visit** (n = 605)		
Yes	530	87.6
No	75	12.4
**Number of ANC visit (n = 605)**		
One	64	10.6
Two	243	40.1
Three	147	24.3
Four and above	151	25
**Partner discussed of HIV test with his wife before ANC visit** (n = 598)		
Yes	496	83
No	102	17
**Partner visited the PMTCT clinic with his wife/wives** (n = 605)		
Yes	500	82.6
No	105	17.4
**Received information from Health care provider**		
On antenatal care visit (n = 500)		
Yes	366	73.2
No	134	26.8
On HIV test (n = 500)		
Yes	468	93.6
No	32	6.4
On PMTCT (n = 449)		
Yes	443	88.8
No	6	11.2
**Sociocultural factors**:		
Pregnancy is the domain of women (n = 605)		
Yes	388	64.1
No	217	35.9
A pregnant woman can be tested for HIV without the husband permission (n = 605)		
Yes	534	88.3
No	71	11.7

### Level of male partner involvement in HTC

More than half (53.7% at 95% CI: 49.6 to 57.5) of male partners were involved in HTC with their wives. Among those involved partners, 365 (60.3%) discussed with their wives about HIV tests and 252 (41.7%) attended ANC visits with their wives (Table 4).

**Table 4:** Level of involvement of male partner of pregnant women towards HIV testing and counseling in Delanta District, Northern Ethiopia 2018.

Variable	Frequency	%
Knowing ANC appointment	279	46.1
Tested for HIV with wife/wives	254	42
Discusses antenatal care HIV testing	365	60.3
Provide financial support	334	55.2
Attend ANC	252	41.7
**Level of male partner involvements score**		
Involved in HTC (4–5 score)	325	53.7
low involvement in HTC (0–3 score)	281	46.3
**Reasons for partners involvement (n = 325)**		
Feel responsibility	254	78.2
Initiated by my wife/wives	245	75.4
Initiated by provider	243	74.8
**Reason of low involvement (n = 281)**		
Fear of being tested	241	85.7
Being busy	239	83
Proxy testing	259	90.2
Neglecting importance	166	57.8
Fear of stigma and discrimination	140	48.8
Away from working	150	52.3
Not my concern	110	38.5

### Factors associated with male partner involvement in HTC

In multivariable logistic regression analysis, male partners living together with pregnant mothers, ever heard about HIV/AIDS from health professionals; male partners discussions with their wives, pregnant mothers ANC visit, and partners who visited PMTCT clinics with their wives were significantly associated with male partner involvement in HTC. Male partners who were living together with their wives were 2.07 times more likely to be involved in HTC than partners who were not living together (AOR: 2.07: CI(1.308–3.275)). Male partners who had discussions with their wives before HTC was 3.72 times more likely to be involved in HTC than male partners who had no open discussions with their wives (AOR 3.72: CI (1.425–6.636)). Besides, male partners who had ever heard about HIV/AIDS from health care providers were 2.08 times more likely to be involved in HTC than male partners who had never heard about HIV/AIDS (AOR: 2.08: CI (1.246–3.486)) (Table 5).

**Table 5:** Bivariate and multivariable analysis of factors associated with male partner involvement in HTC in Delanta District, Northern Ethiopia, 2018.

Variable	Male involvement		COR with 95% CI	AOR with 95% CI
	Yes	No		
Living together				
Yes	276	211	1.91(1.268–2.871)	2.07(1.308–3.275)*
No	48	70	1	
Duration of living together				
1–5 years	107	112	1.19(0.732–1.943)	1.76(1.007–3.085)*
6–10 years	114	83	0.83(0.504–1.365)	1.2(0.682–2.11)
11–15 years	54	43	0.91(0.512–1.609)	1.05(0.552–2.005)
16+years	45	43	1	
Ever heard about HIV/AIDS from health care providers				
Yes	289	221	2.27(1.399–3.671	2.08(1.246–3.486)*
No	30	52	1	
Pregnant women ANC visit				
Yes	306	224	4.33(2.478–7.552)	2.25(1.122–4.517)*
No	18	57	1	
Discussion with their wives				
Yes	268	193	2.18(1.488–3.200)	0.97(0.575–0.99)
No	56	88	1	
Partner visited the PMTCT clinic with his wife				
Yes	285	195	3.22(2.118–4.905)	2.15(1.243–3.716)*
No	39	86	1	
Discussion before the HIV test at home				
Yes	288	36	2.81(1.819–4.334)	3.72(0.575–1.62)*
No	208	72	1	
Disclosure of HIV test result				
Yes	293	212	2.81(1.813–4.347)	0.71(0.310–1.606)
No	31	69	1	

NB

* statistically significant at p-value ≤ 0.05.

## Discussion

This study revealed that 53.7% of partners were involved in HIV testing and counseling (HTC) services provided to women during ANC visits. This level of male partner involvement was higher than the study conducted in South Africa, Fantail district, Bale zone, Lemo district, Harare, and Gondar town [8,9,12,14,20,21] respectively. The discrepancy might be due to time variation; study methods used and study settings, socio-demographic characteristics of the study participants, and availability and accessibility of the infrastructures.

The result of this study was consistent with a study conducted in Arba-Minch [20].

More than 78.2% of male partners were involved in HTC that might be because they feel responsible. Besides 75.4% and 74.4% of male partners were also involved in HTC initiated by their wife’s and health professional’s discussion. However, this finding was lower than the study conducted in Gondar [12].

Regarding the general source of information about HIV/AIDS, this particular study population was obtained HIV-related information from health care providers, the health development army, mass media, wives, relatives, and religious leaders. The major source of information for male partners in this study was health care providers.

In this study, the involvement of male partners was determined by a lack of knowledge, individual, socio-cultural, relationship, and health system factors. Out of the total respondents, 46.3% of male partners never involved in HIV counseling and testing with their wives during ANC follow-up at last pregnancy. This is lower than the study conducted in Gondar, Bale, and Fatale District [12,20,21]. Proxy testing, fear of HIV testing, and work overload were identified as the major reasons for the low involvement of male partners in HTC 90.2%, 85.7%, and 83% respectively. Being busy was a reason for males not to be involved in HTC at the antenatal clinics. Similarly, this finding was similar to a study finding from Tanzania and Bale, Ethiopia. Another reason for men not being involved in this study was Proxy testing that considered their partner’s HIV status was similar to their status. This misconception by men could arise from less access to detailed information on HIV/AIDS messages.

Further barriers recognized in this study were fear of being tested, neglecting importance, and away for work reasons. Fortunately, all low-involved partners knew that the benefits of HIV testing and counseling. This finding is consistent with a study in Blantyre, Malawi [18]. The reasons may be due to good health perception, feeling shame, especially at a young age.

Regarding male partner communications, 83.2% of males reported that their wives have disclosed their HIV status to them during their last pregnancy. This finding is lower than the study found that was reported in a study conducted in Mekelle, Ethiopia [5]. On the other hand, 88.3% of male partners agreed that a pregnant mother should never get permission from her husband for HIV testing and counseling. This finding is lower than the study result that was reported in a study conducted in Bale, Ethiopia [21]. The discrepancy could be due to setting differences and cultural influences. Another possible justification could be cultural barriers that were not addressed in this study.

The involvement of male partners in HIV testing and counseling was found significantly associated. Male partners living together with their wives, ever heard of HIV/AIDS from health professionals, and male partners’ discussions with their wives about HTC, and partner visited PMTCT clinic with their wife found significant factors of the study.

The result of this study showed that male partners who were living together with their wives for 1–5 years were 1.76 times more likely to be involved in HTC than those who were living together for 16^+^years. This study finding is in line with the study finding conducted in South Africa, Oromia-Fantale district (Ethiopia), and Gondar town (Ethiopia) [8,12,21] respectively. This might be due to the shared responsibility among couples living together being able to increase involvement in HTC. In other words, there might be no open discussion on antenatal care intervention, financial support, and the presence of household workload during ANC follow-up will decrease the involvement of male partners.

Regarding pregnant women’s antenatal care visit in a previous pregnancy, it was found 2.25 times more likely to have male involvement in their HTC than those who had no ANC visit. This is similar to the study conducted in Harare and Hadiya Zone, Lemo District [9,14]. This may be because of a positive influence from male engagement-related information and education afforded to pregnant women by healthcare workers.

Uniquely in this study, male partners who have visited ANC/PMTCT clinics with their wives were 2.15 times more likely to be involved in HTC than male partners who were not visited ANC PMTCT clinics in the current pregnancy period. However, this was not observed in other studies. This discrepancy may occur due to study settings and the cultural influence of study areas.

In this study, male partners who have ever heard about HIV/AIDS from a health care provider showed a significant association with male partner involvement. The male partner who had received information about HIV/AIDS were two times more likely to be involved in HTC than those who had not received information. This result was similar to the study conducted in the Meket district, Ethiopia [3]. This might be due to the efforts of health extension workers and the availability of midwives and other health professionals who are assigned to deliver these services to the community.

Male partner discussion with the wife before getting HIV testing and counseling showed a significant association with male partner involvement. In this study, male partners who had discussions at home before getting HTC with their wives were 3.72 times more likely to be involved in HTC than those partners who have no discussions before. This result was supported by a study finding from Mekelle, Ethiopia [19].

## Limitation of the study

This study was conducted at the community level for addressing male partners’ involvement barriers in HTC. However, the possibility of social desirability bias due to sensitiveness of the issue may underestimate male involvement due to using multiple measures of indicators is a limitation of this study. This study did not also include the disclosure status and knowledge aspect of male partners.

## Conclusion

In the district, more than half of male partners were involved in HIV testing and counseling. Male partner involvement was significantly associated with living together of partners, ever heard about HIV/AIDS from health professionals and discussion before getting HTC, wives ANC visit, and partner visited PMTCT clinic with their wives and counseling. The proportion of male partner involvement was found to be low as compared to the national standards. Therefore, local health authorities and health care workers need to develop and conduct interventions that help partners with their wife to live together, improve their awareness about HIV and testing, ANC visit by pregnant women, and encourage having home discussion before HIV testing through counseling raise the level of male partner involvement in HIV testing and counseling.

## Abbreviations

ANC: antenatal care
AOR: adjusted odds ratio
ART: antiretroviral therapy
ARV: antiretroviral disease
BSC: bachelor of science
CI: confidence interval
EDHS: Ethiopian demographic health survey
FGD: focus group discussion
HIV/AIDS: human immunodeficiency virus/acquired immunodeficiency syndrome
HTC: HIV counseling and testing
MPI: male partner involvement
MTCT: mother to child transmission
PMTCT: prevention of mother to child transmission of HIV
SPSS: statistical package for social science
VIF: variance inflation factor

## References

[1] UNAIDS, Fact sheet–global HIV statistics, November 2017.

[2] Joint United Nations Programme on HIV/AIDS (UNAIDS): Prevention of HIV transmission from mother to child Strategic options, Geneva, Switzerland, 1999.

[3] World Health Organization (WHO): Prevention of Mother-To-Child Transmission (PMTCT) brief note: Department of HIV/AID, October 1st, 2007.

[4] Lemma E, Husein G. Male Partner Involvement on Prevention of Mother to Child Transmission of HIV and Associated Factors among Pregnant Mothers Attending Antenatal at Fatale District, Ethiopia. J. Women’s Health Care. 2017; 6(362):2167–0420.

[5] Birhane T, Assefa Tessema G, Addis Alene K, Dadi AF. Knowledge of pregnant women on mother-to-child transmission of HIV in Meket District, Northeast Ethiopia. Journal of pregnancy, 2015. 10.1155/2015/960830 25741447 PMC4337054

[6] Ethiopian public health institution, HIV Related Estimates and Projections for Ethiopia, A.A., March 2017.

[7] Alemu Z, Abebaw G, Lemma D, et al, male Partner’s Involvement in HIV Counseling and Testing and Associated Factors among Partners of Pregnant Women in Gondar Town, Northwest Ethiopia, July 31, 2016. 10.1155/2016/3073908 PMC4983370 27555968

[8] Manami U, A Literature Review on Male Involvement in HIV Testing and Counseling among Pregnant Women in Sub-Saharan Africa, University of North Carolina, Malawi, 2012.

[9] Tilahun and Mohamed S., “male partners’ involvement in the prevention of mother-to-child transmission of HIV and associated factors in Arba Minch Town and Arba Minch Zuria Woreda, Southern Ethiopia,” Biomed Research International, vol. 2015, 2015. 10.1155/2015/763876 PMC4469755 26146631

[10] Motlagabo G. Matseke, Robert A. C. Ruiter, Violeta J. Rodriguez ID, Karl Peltzer ID, Geoffrey Setswe, and Sibusiso Sifunda, Factors Associated with male partner involvement in programs for the prevention of mother-to-child transmission of HIV in Rural South Africa, November 2017. 10.3390/ijerph14111333 PMC5707972 29104275

[11] A factsheet to the WHO consolidated guidelines on HIV testing services. Geneva: World Health Organization; 2015. 26378328

[12] Molla Godif, Huruy Assefa, Mussie Alemayehu, and Wondowosen Terefe, Factors Associated with HIV Counseling and Testing among Males and Females in Ethiopia: Evidence from Ethiopian Demographic and Health Survey, Mekelle University Ethiopia, Data February 20, 2015.

[13] Kinuthia Francis Kariuki1 and Gloria K. Seruwagi, Determinants of Male Partner Involvement in Antenatal Care in Wakiso District, Uganda, November 2016.

[14] Degefa Tadele Belato, Abera BeyamoMekiso, and Bayu Begashaw male Partners Involvement in Prevention of Mother-to-Child Transmission of HIV Services in Southern Central Ethiopia: In Case of Lemo District, Hadiya Zone, 15 March 2017. 10.1155/2017/8617540 PMC5376926 28409027

[15] Delanta Woreda Government communication office 2009 Annual report, Delanta, Wogeltena, 2009 E.C.

[16] Assefa Fekede, Gelato Ayele, Dessie Yadeta, male partner’s involvement in maternal ANC care: The view of women attending ANC in Harare public health institutions, eastern Ethiopia, Vol. 2, No. 3, 2014, pp. 182–188.

[17] Mulusew T, Male involvement in prevention of mother-to-child transition of HIV in the context of partner HIV testing & associated factors at ANC in Goba Town Bale Zone Oromia region, South East Ethiopia Addis Ababa University, May-2015.

[18] Coulibaly Malik, Thio Elisabeth, Yonaba Caroline, Sylvie Ouédraogo Nicolas Meda, Kouéta Fla, et al., “Prevention and care of pediatric HIV infection in Ouagadougou, Burkina Faso: knowledge, attitudes, and practices of the caregivers,” BMC Pediatrics, 16 (1), 2016. 10.1186/s12887-016-0569-y PMC4784410 26961234

[19] Endawoke, T. Gebeyaw, and Amanuel, “Level of male partner involvement and associated factors in the prevention of mother to child transmission of HIV/AIDS services in Debre Marko’s town, Northwest Ethiopia," African Journal of AIDS and HIV Research, 2013, 1 (2), 16–25.

[20] Ekaete Francis, Involvement in Health care research and policy development in the context of mother to child HIV/AIDS transition, Ottawa, Canada 2016.

[21] Haile F., Brian Y. Male partner involvements in PMTCT: a cross-sectional study, Mekelle, Northern Ethiopia. BMC Pregnancy and Childbirth. 2014. 10.1186/1471-2393-14-65 PMC3923985 24521216

